# Highly Norbornylated Cellulose and Its “Click” Modification by an Inverse-Electron Demand Diels–Alder (iEDDA) Reaction

**DOI:** 10.3390/molecules26051358

**Published:** 2021-03-04

**Authors:** Christina Wappl, Viktor Schallert, Christian Slugovc, Astrid-Caroline Knall, Stefan Spirk

**Affiliations:** 1 Institute for Chemistry and Technology of Materials, Graz University of Technology, Stremayrgasse 9, 8010 Graz, Austria; christina.wappl@outlook.com (C.W.); viktor.schallert@tugraz.at (V.S.); slugovc@tugraz.at (C.S.); 2 Institute of Bioproducts and Paper Technology, Graz University of Technology, Inffeldgasse 23A, 8010 Graz, Austria

**Keywords:** cellulose, click chemistry, iEDDA, copper-free, norbornene cellulose, cellulose ester

## Abstract

A facile, catalyst-free synthesis of a norbornylated cellulosic material (NC) with a high degree of substitution (2.9) is presented by direct reaction of trimethylsilyl cellulose with norbornene acid chloride. The resulting NC is highly soluble in organic solvents and its reactive double bonds were exploited for the copper-free inverse-electron demand Diels–Alder (iEDDA) “click” reaction with 3,6-di(pyridin-2-yl)-1,2,4,5-tetrazine. Reaction kinetics are comparable to the well-known Huisgen type 1,3-dipolar cycloaddition of azide with alkynes, while avoiding toxic catalysts.

## 1. Introduction

In recent years, “click” chemistry entered into the focus of research in many disciplines, since it allows for a selective coupling of two or more moieties via dedicated functional groups [1,2]. An ideal “click” reaction leads to selective and fast conversion of the reaction partners, while being environmentally benign. In order to prevent undesired side reactions and by-products, the effort for working up the reaction product is minimized. A well-known “click” reaction is the Cu(I)-catalyzed Huisgen 1,3-dipolar cycloaddition of azides and terminal alkynes (CuAAC) to give 1,2,3-triazoles [1,3]. The popularity of the CuAAC originates from its robustness, tolerating a wide range of chemical conditions as well as good stereoselectivity. “Click” reactions as efficient method of linking two components have made their way into the polymer chemist’s toolbox. Several examples have been also reported for cellulose, the most abundant biopolymer on earth [4,5,6,7,8,9,10]. For materials with an intended use in medicine, in some cases copper is a critical issue and strict regulations do exist due to known side effects on the human body upon excessive copper intake (e.g., liver damage [11], Alzheimer’s disease [12]). As a consequence, intense efforts have been made to establish “click” chemistries which avoid copper catalysts. In this context, it should be mentioned that there are some examples where Huisgen type “click” reactions proceed without any copper catalyst even at room temperature (e.g., cycloadditions of cyclooctynes) albeit reaction kinetics are rather slow and regioselectivity is often not realized in a satisfactory manner [13].

Further copper-free approaches were presented by Bertozzi and Boons [13,14,15,16,17]. However, we became particularly interested in the Diels–Alder reaction with inverse-electron demand (iEDDA) due to the significantly increased reaction kinetics [18] and the possibility to combine iEDDA with other “click” reactions leading to orthogonally modifiable structures [19].

The use of iEDDA reactions between tetrazines and strained olefins and their application as a “click” reaction has not been exploited as extensively as CuAAC, in spite of many impressive applications having emerged recently [20]. Furthermore, the rate constants can be adjusted by selection of appropriate reaction partners, i.e., a less reactive olefin can be converted with a more reactive (more electron-deficient) tetrazine [13,21]. In terms of biocompatibility, tetrazines have been already used in a variety of “click” reactions for an intended use in medicine and a summary of biomedical applications can be found in a review [22,23].

Here, we present a facile approach to “click” cellulose derivatives using an iEDDA reaction avoiding the use of copper. In particular, the reaction of a highly substituted norbornene cellulose ester with a tetrazine is investigated and analyzed using state of the art analytical techniques.

## 2. Results and Discussion

**Figure 1:**
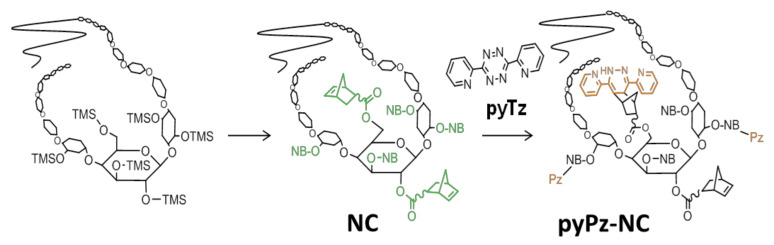
Norbornylation of cellulose and iEDDA reaction on norbornylated cellulose (NC) with 3,6-di(pyridin-2-yl)-1,2,4,5-tetrazine (pyTz) leading to the formation of a dipyridinyl dihydropyridazine (pyPz)-functionalized NC.

Due to the aforementioned good selectivity of iEDDA reactions and their biocompatibility, we were interested in whether we could transfer this concept to cellulose chemistry. To accomplish this target, we identified norbornenes as suitable reaction partners to be immobilized on the cellulose backbone since they are sufficiently reactive dienophiles for iEDDA reactions due to their strain-activated double bond while avoiding the problems typically associated with more reactive dienophiles like trans-cyclooctenes such as storage stability due to isomerization [24]. Another issue is that norbornenes are synthetically easy to access, bearing a variety of functional groups. Consequently, norbornene esters of cellulose were selected as test compounds (Figure 1).

The most convenient route to the desired norbornene cellulose esters proceeds via a catalyst free, direct desilylation-esterification of highly substituted commercially available trimethylsilyl cellulose (TMSC) with norbornoyl chloride in the presence of a base at slightly elevated temperatures [25,26]. Under the chosen reaction conditions, efficient substitution of silyl groups by ester bonds was accomplished as proven for derivatives having a degree of substitution (DS_Si_) of 2.8–2.9. Both the excess acid chloride as well as the exclusive by-product, trimethylsilyl chloride can be easily removed. The reaction gives 2,3,6-norbornoyl cellulose (NC) with a DS close to 3.0 after workup. Workup was performed using a mixture of THF and 0.1 M aqueous K_2_CO_3_ to neutralize traces of nonreacted acid chloride followed by extraction of the NC with dichloromethane. Attempts to replace the THF in the workup by ecologically more favored methanol led to the formation of methyl-5-norbornene 2-carboxylate as side product which is very difficult to separate from the NC. The obtained NC exhibits good solubility in a wide range of organic solvents such as CHCl_3_, toluene, as well as diethyl ether and THF.

**Figure 2:**
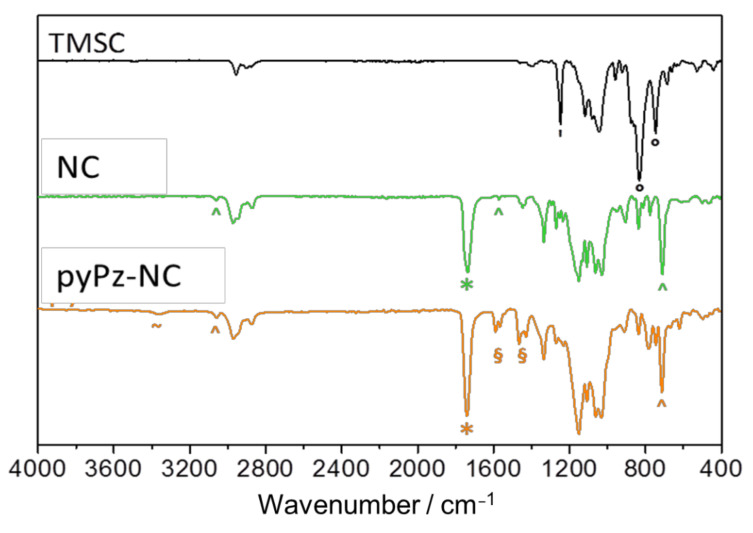
Comparison of the ATR-IR spectra of TMSC, NC and the pyPz-NC. Characteristic bands: ° = TMS groups, ^ = norbornene double bonds * = carbonyl groups, ~ = dihydropyridazine, § = pyridines. Please note that spectra are not normalized.

ATR-FTIR spectroscopy (Figure 2) clearly indicates the formation of the ester bond (*, ν_C=O_ at 1736 cm^−1^) and the presence of norbornene C=C fragments (^, ν_C=C-H_: 711 cm^−1^, ν_C=C_: 1571, ν_C=C-H_: 3061 cm^−1^) concomitant with the disappearance of bands assigned to ν_Si-C_ (‘, 1250 cm^−1^) and δ_Si-O-C_ (°, 833, 746 cm^−1^). 

**Figure 3:**
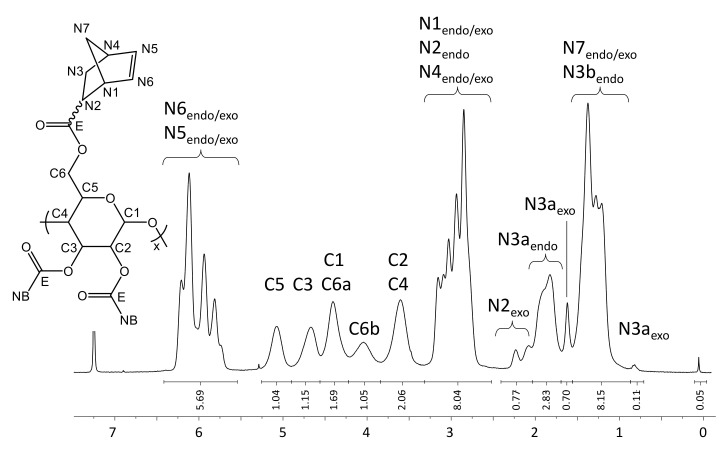
^1^H-NMR spectrum of NC in CDCl_3_ at 298 K.

The presented DS of 2.85 is directly determined from the ^1^H-NMR spectrum (Figure 3). The integral value for the proton signals of the norbornene double bonds (N5 and N6, 6.43–5.55 pm) is related to the integral value of the pyranose ring (C1-C6, 5.27–3.33 ppm). The amount of remaining TMS groups is less than 0.5% as indicated by the small signal at 0.06 ppm.

**Figure 4:**
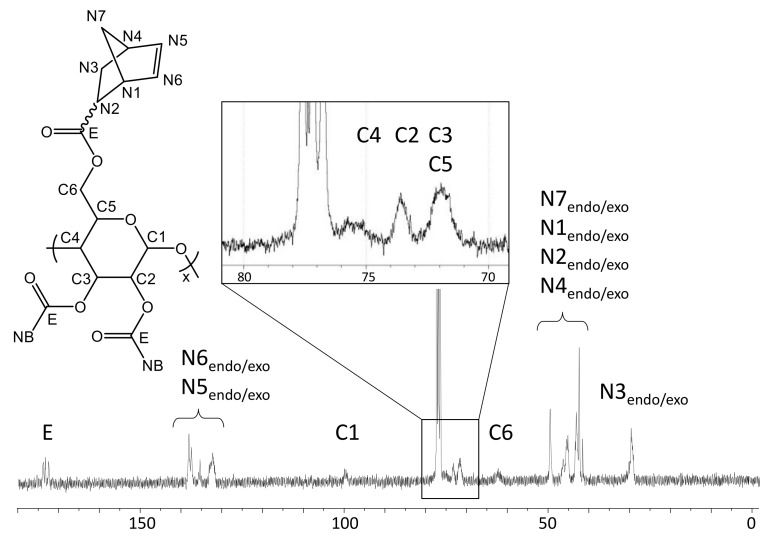
^13^C-NMR spectrum of NC in CDCl_3_ at 298 K.

These assignments are further supported by the ^13^C-NMR spectrum in CDCl_3_ (Figure 4). The Si(CH_3_)_3_ groups present in TMSC (δ: 0.0–1.5 ppm) are below the detection limit of ^13^C-NMR after reaction with norbornene carboxylic acid chloride.

In turn, the typical shifts corresponding to an ester functionality (δ: 172–176 ppm, *endo*- and *exo*-) as well as those corresponding to the norbornene alkenyl carbons (δ: 132.5–138.5 ppm, *endo*- and *exo*-) are observed. There are only isolated signals for each C-atom of the pyranose ring which are, in terms of pattern and chemical shifts, very similar to those reported for cellulose triacetate [27].

**Figure 5:**
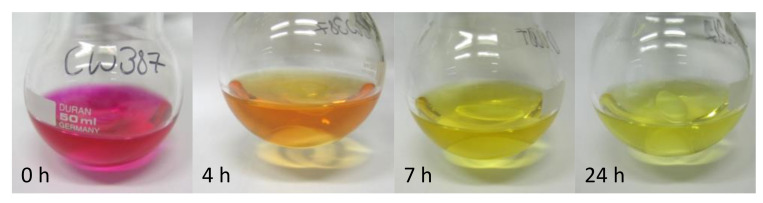
Change in color upon “click” reaction of NC with pyTz.

After synthesis and characterization of the NC, the next step was to explore its capability to participate in an iEDDA reaction with pyTz. After addition of pyTz to NC in a molar ratio of 1:1 (corresponds to a ratio of 1 pyTz per 3 norbornene ester groups), the mixture was stirred overnight at room temperature. The color of the solution changed over this period from pink to yellow as the tetrazine is consumed and the dihydropyridazine (pyPz) moiety is formed (Figure 5). Precipitation of the concentrated solution gave a yellowish solid.

**Figure A4:**
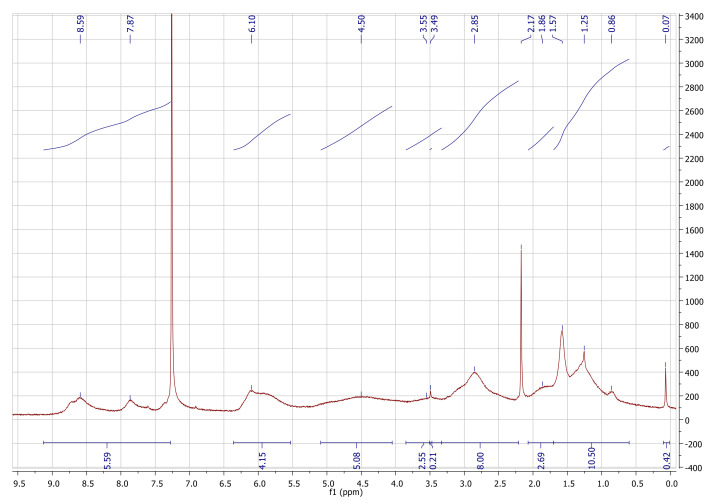
^1^H-NMR spectrum of pyPz-NC in CDCl_3_ at 298 K.

NMR spectroscopy of the pyPz-functionalized NC (pyPz-NC) was attempted, but excessive broadening of the signals hampered a detailed interpretation of the ^1^H-NMR spectrum but indicated the incorporation of the pyPz-moiety into the NC structure by the appearance of broad signals in the aromatic region (see Appendix A, Figure A4). However, the successful “click” immobilization of tetrazines onto NC can be clearly observed in the ATR-FTIR spectra (Figure 2). The new bands between 1400 and 1600 cm^−1^ (§) can be assigned to ring stretching vibrations of 2-monosubstituted pyridines. The signal for N-H stretching vibration at 3370 cm^−1^ (~) confirms the formation of the dihydropyridazine. The signals of the norbornene C=C fragments (^,ν_C=C-H_: 711 cm^−1^_,_ ν_C=C-H_: 3061 cm^−1^) are still visible as statistically a maximum of only 1 out of 2.85 norbornene double bonds per anhydroglucose unit (AGU) are potentially converted.

The product was analyzed additionally by means of elemental analysis (CHN) to estimate the ratio of pyPz moieties on the NC-backbone. The results confirmed the successful “click” transformation introducing approximately 1 pyPz per AGU.

The rate constant *k* of the cycloaddition reaction of NC with pyTz was determined under pseudo first order conditions using UV-Vis spectroscopy. In order to evaluate the reactivity of NC towards pyTz, it was benchmarked with a small molecule, namely 5-norbornene-2-carboxylic acid methyl ester (NM). The distinct absorption peak of the pyTz (λ_max_ = 542 nm in THF) allows for monitoring the progress of the “click” reaction by UV/VIS spectroscopy [28]. In order to ensure pseudo first order reaction conditions, an excess of the norbornene double bonds was accomplished (the norbornene double bond:pyTz ratio was 5:1, 10:1, 15:1) in those experiments (three parallels for each concentration). The pseudo first order reaction rate constants k_app_ at different concentrations were obtained by linear fits of ln([pyTz]/[pyTz_0_]) versus reaction time (Table 1).

**Figure 6:**
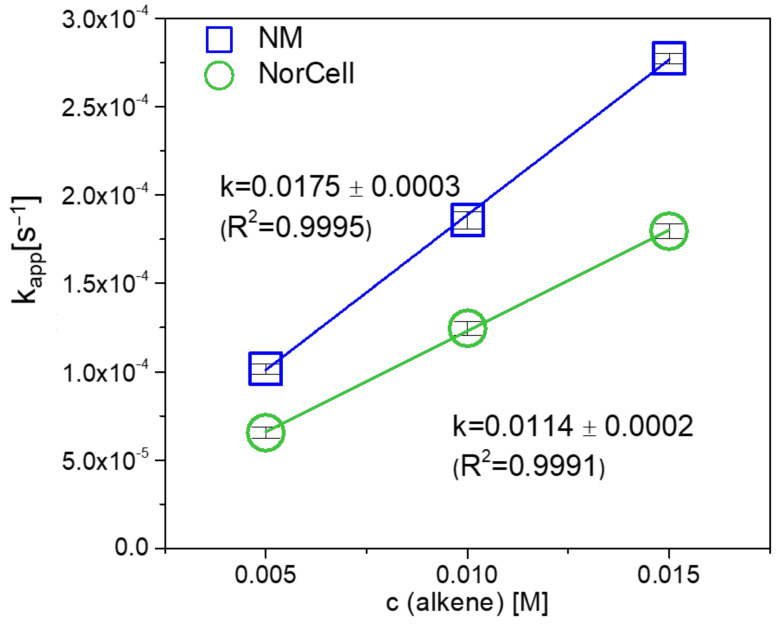
Reaction rates of NC and NM in iEDDA reaction with pyTz in THF under pseudo first order conditions.

Figure 6 depicts the obtained, averaged k_app_ rate constants at different double bond equivalents of NC and NM, respectively. By linear regression, a rate constant for the reaction of NC of 0.0114 mol s^−1^ was derived. This reaction rate only reduced by a factor of 1.5 compared to NM (0.0175 mol s^−1^) which highlights the applicability and versatility of iEDDA reactions particularly for polymeric supports.

## 3. Materials and Methods

### 3.1. Materials

Trimethylsilyl cellulose (TMSC, Avicel, *M*_w_ = 185,000 g·mol^−1^, *M*_n_ = 30,400 g·mol^−1^, PDI = 6.1 determined by GPC in chloroform) with a DS_Si_ value of 2.8–2.9 was purchased from TITK (Rudolstadt, Germany). 5-norbornene-2-carboxylic acid chloride, methyl 5-norbornene-2-carboxylate (NM) and 3,6-di(pyridin-2-yl)-1,2,4,5-tetrazine (pyTz) were prepared according to published literature procedures [29,30,31].

### 3.2. Synthesis

#### 3.2.1. Synthesis of 2,3,6-norbornoyl Cellulose (NC)

TMSC (DS 2.8-2.9, 0.62 g, 1.67 mmol) was added to a mixture of freshly prepared 5-norbornene-2-carbonyl chloride (4.5 g, 28.7 mmol) and 4-dimethylamino pyridine (0.22 g, 1.77 mmol) under an atmosphere of dry nitrogen. After stirring at 80 °C overnight, 20 mL THF were added resulting in the precipitation of 4-dimethylaminopyridine hydrochloride which was removed by filtration. The filtrate was concentrated and added dropwise to an aqueous solution of K_2_CO_3_ (0.1 M) concomitant with the formation of a white solid. After addition of dichloromethane, the organic phase containing the NC was separated, washed again with aqueous K_2_CO_3_ (0.1 M) followed by drying over Na_2_SO_4_. The solution was concentrated in vacuo and added dropwise to cold MeOH. The precipitate was filtered off, washed with cold MeOH and dried in vacuo.

Yield: 0.569 g (67%), colorless powder; DS_NB_ (determined from ^1^H-NMR spectrum of NC in CDCl_3_, Figure 3) 2.85; FT-IR (ATR) [cm^−1^] 3061 (ν_=C-H_); 2972, 2947, 2872 (ν_-C-H_); 1736 (ν_-C=0_); 1571 (ν_-C=C_); 1447 (δ_-C-H_); 1335 (δ_-CH3_); 1296; 1270; 1234, 1151, 1108, 1063 (ν_C-O-C_); 951; 906; 836; 775; 711 (ν_=C-H_); 610; 500; 462; ^1^H-NMR (300 MHz, 298 K, CDCl_3_): δ [ppm] = 6.43–5.55 (N5_endo/exo_, N6_endo/exo_), 5.09 (C5), 4.68 (C3), 4.42 (C1, C6a), 4.05 (C6b), 3.61 (C2, C4), 3.30–2.66 (N1_endo/exo_, N2_endo_, N4_endo/exo_), 2.40–2.05 (N2_exo_), 2.05–1.70 (N3a_endo_), 1.70–1.57 (N3a_exo_), 1.57–0.88 (N7_endo/exo_, N3b_endo_), 0.83 (N3b_exo_); ^13^C-NMR (75 MHz, 298 K, CDCl_3_): δ [ppm] = 174.1–172.6 (E), 138.3–132.5 (N5, N6), 100.2 (C1), 75.7 (C4), 73.4 (C2), 72.0 (C3, C5), 62.6 (C6), 49.8 (N7_endo_), 46.7 (N7_exo_, N1_exo_), 45.5 (N1_endo_), 43.6 (N2_exo_), 43.3 (N2_endo_), 42.7 (N4_endo_), 41.8 (N4_exo_), 30.6 (N3_exo_), 29.9 (N3_endo_); elemental analysis (CHN) [%] 66.52 C, 6.29 H.δ

#### 3.2.2. Synthesis of pyPz-NC via iEDDA

NC (36.4 mg, 0.071 mmol, DS 2.85) was dissolved in THF and a solution of pyTz (20.2 mg, 0.0784 mol, 1.2 eq/AGU) in THF was added quickly. The reaction was stirred at room temperature overnight. A change of color from pink to orange after 4 h and bright yellow overnight was observed characteristic for the conversion of the tetrazine. The solution was concentrated and added dropwise to cold MeOH. The yellowish precipitate was filtered off, washed with cold MeOH and dried under vacuo.

Yield: 29.9 mg (59%), yellowish solid, DS_pyPz_ (determined from elemental analysis) 0.75; FT-IR (ATR) [cm^−1^] 3360 (ν_N-H_); 3058 (ν_=C-H_), 2970 (ν_-C-H_); 1740 (ν_-C=0_); 1589, 1564, 1467, 1428 (ν_2-monosubstituted pyridine_); 1336 (δ_-CH3_); 1271, 1152, 1030 (ν_C-O-C_); 783; 746; 665; 620; elemental analysis (CHN) [%] 66.20 C, 5.76 H, 5.93 N.

### 3.3. Determination of the Rate Constant of the iEDDA Reaction

The rate constants *k* of the iEDDA reaction of NC and NM with pyTz were measured using pseudo first order conditions (constant tetrazine concentration and excess of norbornene) using UV-Vis spectroscopy [28].

Stock solutions of NC (14.0 mM, DS 2.85, i.e., 40.0mM norbornene double bond), NM (40.0 mM) and pyTz (2.0 mM) were prepared in THF. For each experiment, the solutions were mixed in the order NC/NM, THF, pyTz in a quartz cuvette (Table 2) and immediately inserted into a UV-Vis spectrometer. The final concentration of pyTz was 1.0 mM and of norbornene double bonds 5.0, 10.0 and 15.0 mM, corresponding to 5–15-fold excess. The decay of the absorption of pyTz at 542 nm was recorded (1 Hz, 5 min) and the experiments were repeated three times.

### 3.4. Measurements

#### 3.4.1. NMR Spectroscopy

NMR measurements were performed on a Bruker Avance 300 NMR (Billerica, MA, USA) spectrometer. Deuterated solvents were obtained from Cambridge Isotope Laboratories Inc. (Tewksbury, MA, USA) and remaining peaks were referenced according to literature [32]. Peak shapes are specified as follows: bs (broad singlet) and m (multiplet).

#### 3.4.2. Infrared Spectroscopy (ATR-FTIR)

IR spectra were attained by an Alpha FT-IR spectrometer (Bruker; Billerica, MA, USA) using an attenuated total reflection (ATR) attachment. Spectra were obtained in a scan range between 4000 and 400 cm^−1^ with 48 scans and a resolution of 4 cm^−1^. The data was analyzed by Bruker’s OPUS 4.0 software (Bruker; Billerica, MA, USA).

#### 3.4.3. Elemental Analysis

The mass fractions of carbon, hydrogen and nitrogen were determined with a Vario EL III Element Analyzer from Elementar GmbH. (Langenselbold, Germany). The degree of substitution for the clicked products was calculated according to adapted equations from literature [33].

#### 3.4.4. UV/Vis Spectroscopy

Absorption spectra were recorded on a Shimadzu spectrophotometer UV-1800. (Kyoto, Japan) The emission was measured on a Hitachi F-7000 fluorescence spectrometer (Tokyo, Japan) equipped with a red-sensitive photomultiplier R928 from Hamamatsu (Hamatsu, Japan).

## 4. Conclusions

In summary, simultaneous desilylation-esterification of trimethylsilyl cellulose was proven to be a highly efficient way to obtain norbornylated cellulose with a DS > 2.8. The highly substituted NC derivative was subjected to inverse electron-demand Diels–Alder “click” chemistry with 3,6-di(pyridin-2-yl)-1,2,4,5-tetrazine. It turns out that the observed reaction kinetics are competitive with CuAAC while being copper and catalyst free. This procedure could be the starting point for many other reactions involving polysaccharide esters and tetrazines since the presented approach is generic and applicable to many other substrates. Since copper is avoided in this “click” reaction, compounds intended for medical use are a major field where this reaction type could find a large application area [22]. Still, more efforts need to be performed to fully elucidate the structure and substitution patterns of the clicked product. This could be accomplished using solid state ^13^C-NMR spectroscopy and/or isotope labeling (^13^C, ^15^N) of the tetrazine component. 

NC itself is a potentially interesting component for chemical transformations such as grafting-from polymerization using ring-opening metathesis polymerization (ROMP) [34] which opens up a manifold of possibilities to prepare biocomposites, and hybrid materials [35,36]. As opposed to previous endeavors [37], the aforementioned good organosolubility of NC as presented herein enables a true grafting-from approach which could be utilized e.g., for the preparation of bottlebrush copolymers.

## Figures and Tables

**Table 1:** Pseudo first order rate constants k_app_ for iEDDA of pyTz with NC and NM.

c(Alkene) (M)	Pseudo First Order Rate Constant k_app_ (s^−1^)	
	NC	NM
0.005	6.55 × 10^−5^ ± 3.37 × 10^−6^	1.02 × 10^−4^ ± 2.90 × 10^−6^
0.010	1.26 × 10^−4^ ± 3.96 × 10^−6^	1.86 × 10^−4^ ± 4.83 × 10^−6^
0.015	1.79 × 10^−4^ ± 4.10 × 10^−6^	2.77 × 10^−4^ ± 2.79 × 10^−6^

**Table 2:** Mixing formula for alkene:Tz ratios 5:1, 10:1 and 15:1.

Alkene:Tz	NC/ NM (µL)	THF (µL)	pyTz (µL)
5:1	250	750	1000
10:1	500	500	1000
15:1	750	250	1000

**Figure A1:**
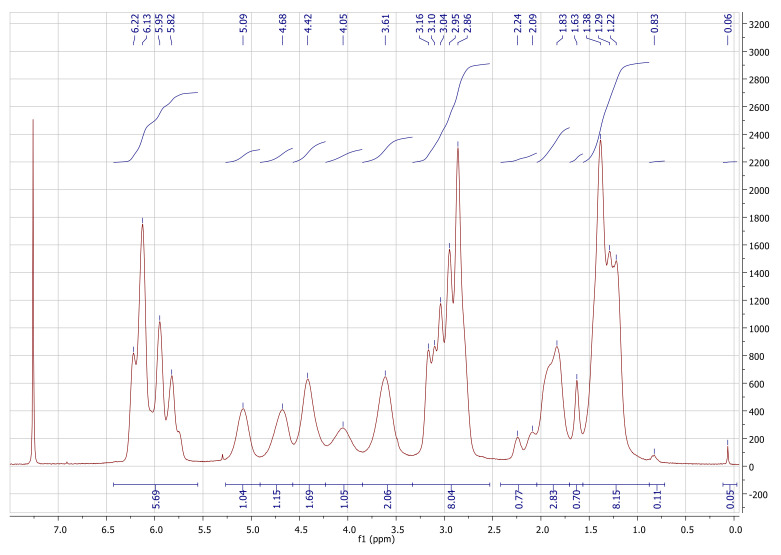
^1^H-NMR spectrum of NC in CDCl_3_ at 298 K.

**Figure A2:**
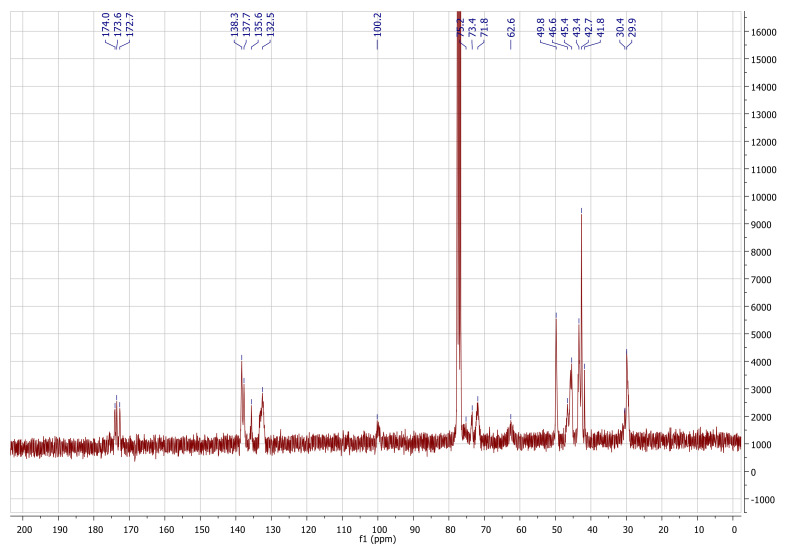
^13^C-NMR spectrum of NC in CDCl_3_ at 298 K.

**Figure A3:**
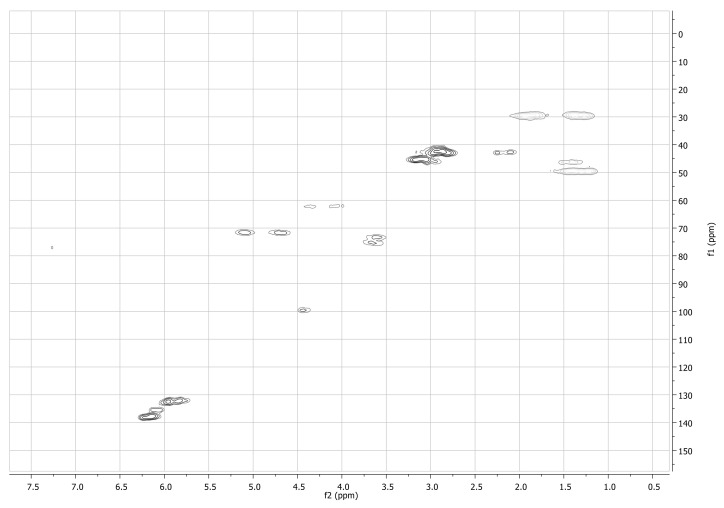
^13^C-^1^H-HSQC NMR spectrum of NC in CDCl_3_ at 298 K.

## Data Availability

Data is contained within the article.

## Sample Availability

Samples of the compounds are available from the authors.

## Appendix A

### Appendix A.1. Characterization Data for NC

**DS** (from ^1^H-NMR spectrum of NC in CDCl_3_, Figure 1): 2.85.

**^1^H-NMR** (300 MHz, 298 K, CDCl_3_): δ [ppm] = 6.43–5.55 (m, 5.69 H, N5_endo/exo_, N6_endo/exo_), 5.09 (bs, 1.04 H, C5), 4.68 (bs, 1.15 H, C3), 4.42 (bs, 1.69 H, C1, C6a), 4.05 (bs, 1.05 H, C6b), 3.61 (bs, 2.06 H, C2, C4), 3.30–2.66 (m, 8.04 H, N1_endo/exo_, N2_endo_, N4_endo/exo_), 2.40–2.05 (m, 0.77 H, N2_exo_), 2.05–1.70 (m, 2.83 H, N3a_endo_), 1.70–1.57 (m, 0.70 H, N3a_exo_), 1.57–0.88 (m, 8.15 H, N7_endo/exo_, N3b_endo_), 0.83 (m, 0.11 H, N3b_exo_), 0.06 (s, 0.05 H, TMS).

**^13^C-NMR** (75 MHz, 298 K, CDCl_3_): δ [ppm] = 174.1–172.6 (C_q_, E), 138.3–132.5 (N5, N6), 100.2 (C1), 75.7 (C4), 73.4 (C2), 72.0 (C3, C5), 62.6 (C6), 49.8 (N7_endo_), 46.7 (N7_exo_, N1_exo_), 45.5 (N1_endo_), 43.6 (N2_exo_), 43.3 (N2_endo_), 42.7 (N4_endo_), 41.8 (N4_exo_), 30.6 (N3_exo_), 29.9 (N3_endo_).

**FT-IR (ATR)** [cm^−1^]: 3061 (ν_=C-H_); 2972, 2947, 2872 (ν_-C-H_); 1736 (ν_-C=0_); 1571 (ν_-C=C_); 1447 (δ_-C-H_); 1335 (δ_-CH3_); 1296; 1270; 1234, 1151, 1108, 1063 (ν_C-O-C_); 951; 906; 836; 775; 711 (ν_=C-H_); 610; 500; 462.

**Elemental analysis** (CHN) [%]: 66.52 C, 6.29 H (measured); 66.52 C, 6.69 H, (calculated with 5 water molecules, NC DS 2.85).

### Appendix A.2. Characterization of pyPz-NC

**DS** (from elemental analysis): 0.75.

**^1^H-NMR** (300 MHz, 298 K, CDCl_3_): δ [ppm] = 8.98–7.29 (m, 5.59 H, py), 6.43–5.55 (m, 4.15 H, N5_endo/exo_, N6_endo/exo_), 5.09–4.05 (m, 5.08 H, C5, C3, C1, C6), 3.85–3.33 (m, 2.55 H, C2, C4, MeOH), 3.30–2.21 (m, 8.00 H, N1_endo/exo_, N2_endo/exo_, N4_endo/exo_), 2.40–2.15–1.70 (m, 2.69 H, N3a_endo_), 1.70–0.60 (m, 10.50 H, N3a_exo_N7_endo/exo_, N3b_endo/exo_, H_2_O), 0.07 (s, 0.42 H, TMS).

**FT-IR (ATR)** [cm^−1^]: 3360 (ν_N-H_); 3058 (ν_=C-H_), 2970 (ν_-C-H_); 1740 (ν_-C=0_); 1589, 1564, 1467, 1428 (ν_2-monosubstituted pyridine_); 1336 (ν_-CH3_); 1271, 1152, 1030 (ν_C-O-C_); 783; 746; 665; 620.

**Elemental analysis** (CHN) [%]: 66.20 C, 5.76 H, 5.93 N (measured); 67.09 C, 6.09 H, 5.69 N (calculated with 5 water molecules, NC DS 2.85, pyPz DS 0.75).
